# Trends and Disparities in *Clostridioides difficile* Infection Mortality in the United States From 1999 to 2020: A Nationwide Perspective

**DOI:** 10.1155/grp/9981233

**Published:** 2026-05-12

**Authors:** Mohamed H. Eldesouki, Abdul-rahman Abusalim, Mohammad Kloub, Muhammed Umer, Mohamed Ahmed Ali, Ahmed Yasser Shaban, Ahmed Salem, Anas Almoghrabi, Jihad Slim, Yatinder Bains, Theodore Dacosta

**Affiliations:** ^1^ Department of Internal Medicine, New York Medical College at Saint Michael’s Medical Center, Newark, New Jersey, USA; ^2^ Department of Internal Medicine, Division of Hospital Medicine, University of Wisconsin School of Medicine and Public Health, Madison, Wisconsin, USA, uwm.edu; ^3^ Qena Faculty of Medicine, Qena University, Qena, Egypt; ^4^ Department of Internal Medicine, Faculty of Medicine, Kafrelsheikh University, Kafrelsheikh, Egypt, kfs.edu.eg; ^5^ Department of Internal Medicine, Maimonides Medical Center, Brooklyn, New York, USA, maimonidesmed.org; ^6^ Department of Internal Medicine, John H. Stroger Jr. Hospital of Cook County, Chicago, Illinois, USA, cookcountyhhs.org; ^7^ Division of Infectious disease, New York Medical College at Saint Michael’s Medical Center, Newark, New Jersey, USA; ^8^ Division of Gastroenterology and Hepatology, New York Medical College at Saint Michael’s Medical Center, Newark, New Jersey, USA

**Keywords:** *Clostridioides difficile infection*, CDC WONDER, *Mortality*, disparities, nationwide perspective

## Abstract

**Background:**

*Clostridioides difficile* infection (CDI) is one of the most prevalent infections in the United States, with rising mortality and disease severity in the early 2000s, followed by a steady decline after 2010. This study analyzes national trends in CDI mortality across different demographic and regional groups from 1999 to 2020.

**Methods:**

We used CDC WONDER (Wide‐ranging Online Data for Epidemiologic Research) to analyze CDI‐related deaths among adults aged ≥ 35 years. Age‐adjusted mortality rates (AAMRs) per 100,000 individuals, along with annual percent change (APC) and average annual percent change (AAPC), were calculated and stratified by year, sex, race, and region. Statistical significance was assessed using Joinpoint regression analysis.

**Results:**

During the study period, 191,653 CDI‐related deaths were reported. The AAMR for CDI‐related mortality increased from 1.1 in 1999 to 7.3 in 2011 (AAPC: 33.60). However, from 2011 to 2020, there was a notable decline in CDI mortality (AAPC: −10.48; 95% CI: −13.31 to −7.52). Mortality increased significantly with age, and females had a higher total number of deaths, while males maintained a higher AAMR throughout the study period. Among different racial groups, non‐Hispanic White individuals recorded the highest AAMR (6.1) in 2011, while Asian individuals had the lowest AAMR (2.3). Geographically, the Northeastern census region reported the highest AAMR (4.8) in 2008, while the Southern census region had the lowest AAMR (3.1). The majority of CDI‐related deaths (78%) occurred in hospitals, with nursing homes accounting for 17.7% of fatalities.

**Conclusions:**

The sharp decline in CDI mortality starting in 2010 aligns with the implementation of strict guidelines and collaborative efforts between professional societies (IDSA/SHEA), the ACG, and government agencies (CDC). This underscores the success of coordinated public health initiatives in reducing healthcare‐associated infections and improving patient outcomes. However, disparities in mortality rates persist across different age, sex, ethnic, and geographic groups, emphasizing the need to further study disease trends and explore strategies to address these disparities in high‐risk populations.

## 1. Introduction

*Clostridioides difficile* infection (CDI) is a major global healthcare challenge, characterized by a high propensity for recurrence, morbidity, and mortality [1]. CDI is caused by *Clostridioides difficile* (*C. difficile*), which is an anaerobic, Gram‐positive, toxin‐producing bacterium [2, 3]. The spores of *C. difficile* are found in healthcare facilities, the environment, and the food supply. They are able to survive for several months in the environment and are transmitted by the fecal‐oral route [2–4]. In 1935, *C. difficile* was first isolated from the feces of breastfed infants, and in 1978, the first CDI was reported as the cause of pseudomembranous colitis [5]. CDI symptoms vary from life‐threatening to asymptomatic. Many CDI patients exhibit symptoms such as fever, abdominal pain, and watery diarrhea [2]. Complications such as colon perforation, septicemia, toxic megacolon, and death may occur in severe cases [2]. Specifically, CDI affects immunocompromised individuals, the elderly, and females at higher rates [6]. Other risk factors for CDI include prolonged use of antibiotics, proton pump inhibitors (PPIs), gastrointestinal surgery, multiple underlying diseases, renal insufficiency, hypoalbuminemia, long hospital stays, and the presence of an indwelling nasogastric tube [3–5].

CDI is a prevalent complication in cirrhotic patients due to frequent antibiotic use, hospitalizations, and immune dysfunction [2]. The CDI mortality rate is nearly 5%, while the mortality rate related to CDI‐associated complications is approximately 15%–25%, and in patients who require intensive care, it rises to 34% [2]. In developed countries, *C. difficile* is one of the major causes of nosocomial infection, with a cumulative incidence rate ranging from 1 to 632 per 100,000 persons per year [2]. In the United States, CDI incidence has not remained the same over the past years; there was a decline from 2011 to 2017 by 24%, with nearly 462,100 cases in 2017 [7]. Healthcare‐associated infections decreased by 36%, and hospitalization periods also decreased; nonetheless, the rates of community infection did not change significantly [7]. The 2021 report of the Centers for Disease Control and Prevention (CDC) for the Emerging Infections Program for CDI estimated that the crude incidence rate in the United States was 110.2 cases per 100,000 persons, with a slightly increased incidence in community infections at 55.9 cases per 100,000 persons, compared with healthcare‐associated infections at 54.3 cases per 100,000 persons [7].

CDI is widely prevalent and continues to gain more importance due to increasing healthcare expenditures [7]. A meta‐analysis of studies between 2005 and 2015 estimated that CDI costs the healthcare system in the United States around $21,448 per case, and the overall cost of managing inpatient CDI was $6.3 billion in 2015 [7]. The standard management of CDI includes antibiotic therapy, such as oral fidaxomicin or vancomycin, for the initial episodes of CDI [4]. Despite these therapies, the recurrence of CDI remains a significant challenge, with nearly 35% or more of treated patients experiencing recurrence [4]. Understanding the broader impact of CDI, including mortality trends and associated disparities, is crucial for improving outcomes and reducing its burden. In this study, we aim to investigate the CDI mortality trends in the United States from 1999 to 2020 and the disparities associated with them across different sexes, races, ethnicities, places of death, and census regions.

## 2. Methods

### 2.1. Selection and Data Collection

We conducted a retrospective longitudinal analysis using data obtained from the Centers for Disease Control and Prevention’s Wide‐ranging Online Data for Epidemiologic Research (CDC WONDER). This online database contains national mortality and population data in the United States. The database is encoded based on death certificates of US residents from January 1, 1999, to December 31, 2020, and contains information on the underlying and multiple causes of death, along with demographic data. International Classification of Diseases, 10th Revision (ICD‐10) was used to classify the causes of death for 1999 and beyond.

Using the CDC WONDER from 1999 to 2020, we used ICD‐10 (A04.7) to identify individuals who had CDI as an underlying cause of death in their death certificates. Individuals younger than 35 years old at the time of death (based on the death certificate) were excluded from the data query. This study approach has been validated in similar research on other topics of interest.

We first evaluated the age‐adjusted mortality rate (AAMR; standardized to 2000 US Census proportions) related to the CDI in the general population. Then, we stratified the AAMR based on age, sex, race, and geographic region of residence. Furthermore, we reported the mortality percentages for 10 age groups and in different places of death. Our study did not require institutional review board approval because the population data are deidentified and publicly available.

### 2.2. Statistical Analysis

We obtained the AAMR for overall CDI, stratified by sex, directly from the CDC WONDER database and charted the trends throughout the study period. The AAMR per 100,000 was calculated using the direct method by applying age‐specific rates in a population of interest to the 2000 US Standard Population. This allows for the reduction of confounding effects due to varying age structures and enables meaningful comparisons across different populations. We used the Joinpoint regression program (Joinpoint V4.9.1.0; National Cancer Institute) to evaluate trends of AAMR in each subgroup.

This method determines the significance of AAMR changes over time using log‐linear regression models where temporal variation occurred. Annual percent change (APC) with 95% CI for the AAMR was calculated using the Monte Carlo permutation test at the identified line segments linking Joinpoint. Afterward, the weighted averages of the APCs, also known as average annual percent change (AAPC), were calculated with corresponding 95% CI, which reflects the summary of the mortality trends in the study period. Statistical significance was set at *p* ≤ 0.05 using a 2‐tailed *t*‐test. The population was further categorized into large, fringe metro (indicated as urban) and noncore, nonmetro counties (rural) according to the 2013 US Census classifications.

## 3. Results

### 3.1. Overall Results and Sex‐Based Results

**Figure 1:**
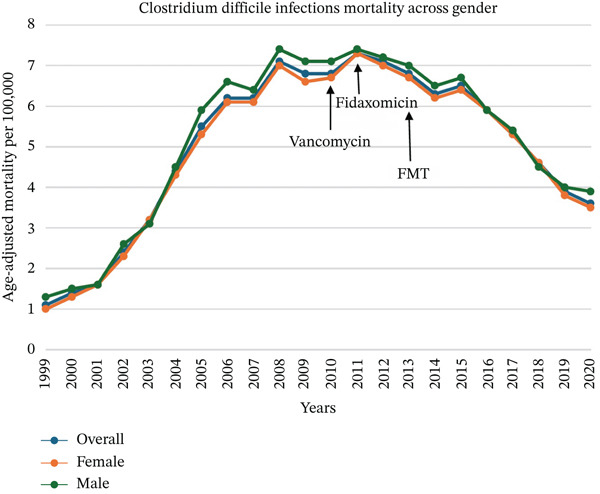
Trends in age‐adjusted mortality rates of *Clostridioides difficile* infections between 1999 and 2020.

Individuals aged 35 years and above were included in the analysis, with 191,653 deaths attributed to CDI in the United States during the study period. The AAMR for CDI‐related deaths increased significantly from 1.1 per 100,000 in 1999 to its peak (7.3) in 2011; then, it decreased to 3.6 in 2020 (Figure 1). The APC of 33.60% (95% CI: 30.45–37.76, *p* value < 0.01) from 1999 to 2008 was followed by a downward trend with an APC of −10.48% (95% CI: −13.31 to −7.52, *p* < 0.01) through 2020 (Figure S1).

In terms of sex, males recorded 79,243 deaths (41.35%), while females had a higher number of deaths of 112,410, with a percentage of 58.65%. Although males had a slightly higher AAMR than females throughout the study period, females exhibited a higher APC. The AAMR in males increased from 1.3 in 1999 to a peak of 7.3 in 2011, with an APC of 18.58% (95% CI: 12.87–32.57, *p* < 0.01), followed by a marked decline, with the AAMR dropping to 3.9 in 2020, accompanied by an APC of −4.7% (95% CI: −7.4 to −2.7, *p* < 0.01) (Figure 1 and Figure S1). Similarly, females experienced a rise in AAMR from 1.0 in 1999 to 7.2 in 2011, with an APC of 25.93% (95% CI: 24.77–27.01, *p* < 0.01), with a decline thereafter until AAMR reached 3.5 in 2020, with an APC of −4.82% (95% CI: −7.46 to −4.7, *p* < 0.01) (Figure 1 and Figure S1).

### 3.2. Mortality Over Age Groups

**Figure 2:**
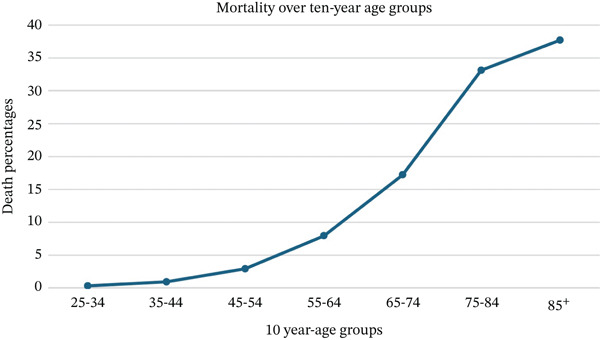
Death percentages over 10‐year age groups of *Clostridioides difficile* infections between 1999 and 2020.

The mortality from CDI increased significantly with age. The percentages of deaths in the study populations under 45 years old were less than 1%; however, these mortality rates rose steadily. The individuals who were aged between 35 and 44, 45 and 54, 55 and 64, and 65 and 74 years had percentages of 0.91%, 2.91%, 7.91%, and 17.2%, respectively. The highest mortality percentages were observed in individuals 75 years and older, where the proportion of deaths was 33.11%, and this percentage increased to 37.71% in individuals who were aged > 85 (Figure 2).

### 3.3. Race/Ethnicity

**Figure 3:**
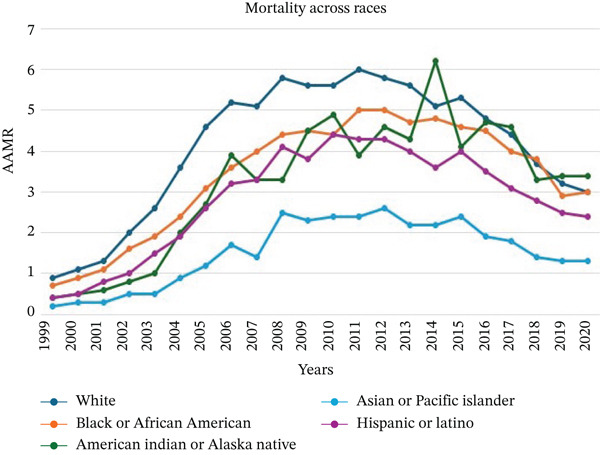
Trends in age‐adjusted mortality rates of *Clostridioides difficile* infections across different races between 1999 and 2020.

Over the study period, AAMR throughout racial groups was different. The non‐Hispanic (NH) Whites had the highest AAMR at 6.1. This was followed by the NH African Americans at 5.1 and the American Indian or Alaska Native at 5. The Hispanic and Latino group recorded an AAMR of 3, and lastly, the NH Asian or Pacific Islander group had the lowest mortality of 2.3 (Figure 3).

The White population had an increase in the AAMR from 1999 (0.9) until 2008 (5.6), with an APC of 4.49 (95% CI: 0.45–10.25, *p* < 0.01), followed by a decline to 3 in 2020, with an APC of ‐7.71 (95% CI: −8.240 to − 7.27, *p* < 0.01). For the African Americans, the AAMR started at 0.7 in 1999, then increased to 5.0 in 2011. The APC was 7.43 (95% CI: 4.46–12.43, *p* < 0.01) from 1999 to 2008 and later decreased to −7.04 from 2009 to 2020 (95% CI: −9.4 to −4.96, *p* < 0.01). Hispanic or Latino individuals recorded AAMR at 0.4 in 1999, which increased to 4.4 in 2010, showing an APC of 9.48 (95% CI: 6.26–14.39, *p* < 0.01); however, the AAMR declined to 2.4 in 2020, with an APC of −7.09 (95% CI: −8.82 to −5.60, *p* < 0.01).

American Indian individuals had an AAMR of 0.8 in 2002, which increased to 6.5 in 2014, with an APC of 7.19 (95% CI: 2.97–121.84, *p* < 0.01); however, it decreased to 3.4 in 2020 with an APC of −9.08 (95% CI: −31.84 to −1.058, *p* < 0.01). Finally, in the Asian or Pacific Islander groups, the AAMR was 0.5 in 2002, which increased to 2.6 in 2012, followed by a decrease to 1.3 in 2020. The APC was 9.48 (95% CI: 6.26–14.39, *p* < 0.01) from 2002 to 2009, then decreased to −7.09 (95% CI: −8.82 to −5.60, *p* < 0.01) from 2009 to 2020 (Figure 3 and Figure S2).

### 3.4. Census Region and State‐Wise

**Figure 4:**
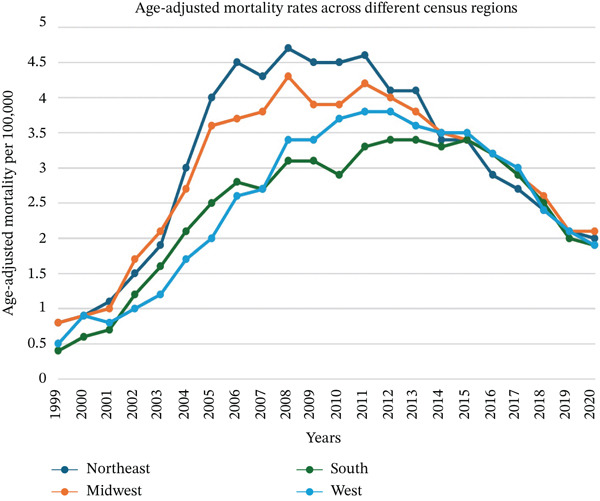
Trends in age‐adjusted mortality rates of *Clostridioides difficile* infections across different census regions between 1999 and 2020.

**Figure 5:**
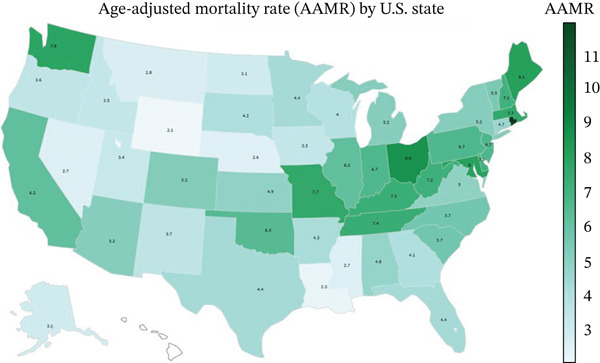
Age‐adjusted mortality rates across the US different states.

**Figure 6:**
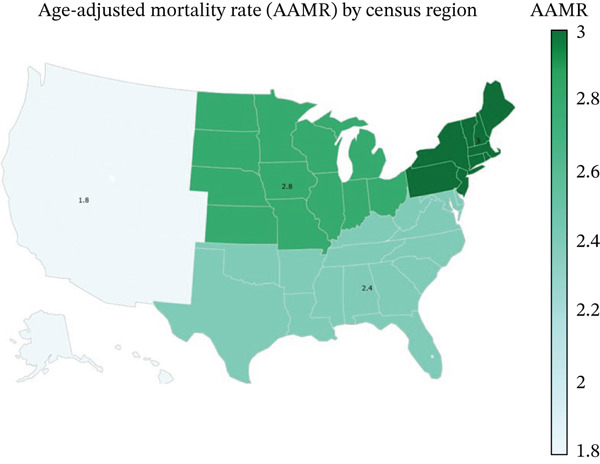
Age‐adjusted mortality rates across the US different regions.

Over the study period, notable differences in the mortality rates were observed across multiple regions and states within the United States. The Northeast region had the highest AAMR at 4.8, followed by the Midwest region at 4.3, the West region at 3.7, and the South region with the lowest AAMR at 3.1. At the state level, Rhode Island recorded the highest AAMR of 8.50, followed by Ohio, with an AAMR of 6.40. States in the Top 10th percentile of high mortality included Delaware, Maine, Maryland, Missouri, and New Hampshire, while Hawaii and Louisiana recorded the lowest AAMR of 1.0 and 2.0, respectively. States in the bottom 10th percentile of AAMR included Mississippi, North Dakota, Alaska, Idaho, Hawaii and Louisiana" (Figures 4, 5, and 6).

### 3.5. Urban and Rural Areas

**Figure 7:**
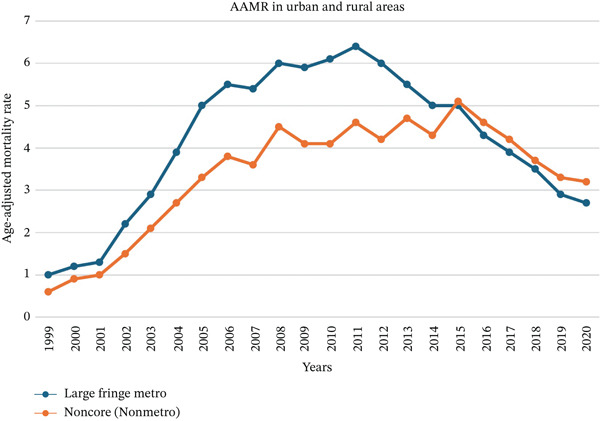
Trends in age‐adjusted mortality rates of *Clostridioides difficile* infections across urban and rural areas between 1999 and 2020.

Overall, the AAMR due to CDI was higher in urban areas (large metropolitan) (4.2) compared to rural areas (nonmetropolitan) (3.4); however, the AAMR in rural areas (3.2) became higher than the urban ones (2.7) in 2020. In urban areas, AAMR was 1.0 in 1999, rising to 6.4 by 2011, before declining to 2.7 in 2020. In contrast, rural areas had an AAMR of 0.6 in 1999, peaking at 5.1 in 2015, then slightly decreasing to 3.2 in 2020 (Figure 7). From 1999 to 2005, the AAMR sharply increased in terms of mortality with an APC of 33.69 (95% CI: 29.15–40.42, *p* < 0.01) in the urban areas and 32.65 (95% CI: 28.56–38.69, *p* < 0.01) in the rural areas, followed by a mild increase with an urban APC of 4.11 (95% CI: 2.29–5.7, *p* < 0.01) and rural APC of 3.46 (95% CI: 2.23–4.78, *p* < 0.01) through 2015. After 2015, the AAMR had a decreasing APC of −8.47 (95% CI: −9.7 to −7.48, *p* < 0.01) in urban areas and −8.93 (95% CI: −12.33 to −6.42, *p* < 0.01) (Figure S4).

### 3.6. Place of Death

**Figure 8:**
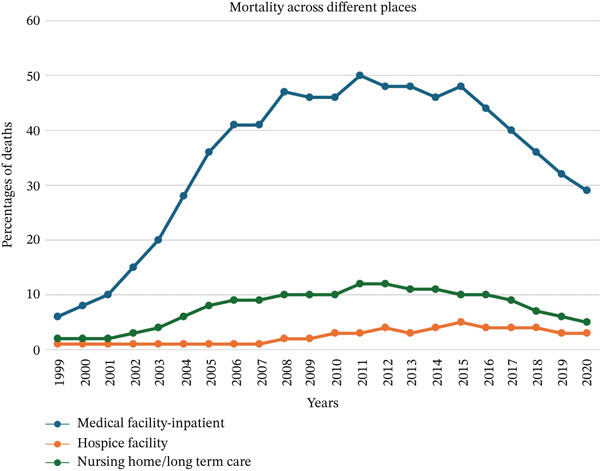
Death percentages in different places of deaths due to *Clostridioides difficile* infections between 1999 and 2020.

Most CDI‐related deaths (78%) occurred in the hospitals. Nursing homes accounted for 17.7%, while hospice facilities were responsible for 4.3% of the CDI mortality (Figure 8).

## 4. Discussion

Utilizing data extracted from the CDC WONDER database, this research provides a comprehensive analysis of CDI‐related mortality in the United States from 1999 through 2020. Our results show significant variations in mortality rates throughout the years, with notable demographic trends and disparities. A total of 191,653 CDI deaths were reported during the study period. The AAMR for CDI‐related mortality increased from 0.6 in 1999 to 7.3 in 2011. AAMR significantly declined afterwards with an AAPC of −10.48. The NH White individuals recorded the highest AAMR (6.1) while the Asian individuals had the lowest AAMR (2.3). The Northeastern region had the highest AAMR of 3, while the Southern census region had the lowest one (2.8). The findings of our study demonstrated a marked increase in AAMR for CDI between 1999 and 2011, followed by a steady decline through 2020. The peak AAMR of 7.3 in 2011 was followed by a steady decline to 3.6 in 2020.

To understand the justification behind these results, we must look in‐depth at the advancements in our understanding and approach to epidemiology, diagnosis, and management of *C. difficile* colitis over the years, with emerging FDA approvals of new medications and updated guidelines from both Infectious Diseases Society of America/Society for Healthcare Epidemiology of America (IDSA/SHEA) and American College of Gastroenterology (ACG) from 1995 to 2021.

Epidemiologically, the prevalence of CDI was comparatively constant throughout the late 1990s. Following that, the prevalence of CDI began to increase in 2000 and peaked in 2010, when it was ranked as the 18th most common cause of death for people aged 65 and over. BI/NAP1/027, a novel strain of *C. difficile*, was one of the reasons behind this rise. It has been demonstrated to have several characteristics that contribute to its hypervirulence, including higher binding capacities, greater toxin synthesis, enhanced motility, and antimicrobial resistance. Because of its capacity to alter its genetic makeup, *C. difficile* was able to evolve into this more pathogenic strain [8–10]. Furthermore, the increasing use of antibiotics during that time before starting the antibiotic stewardship programs led to this breakout of the CDI and mortality peaks. Looking into guidelines, the initial 1995 IDSA/SHEA guidelines focused on hospital‐associated CDI, with little attention to other epidemiological sources. It relied mainly on clinical diagnosis and toxin A/B assays. Metronidazole was the first‐line treatment for mild to moderate cases, with limited options for severe CDI. There was little to no implementation of antibiotic stewardship or advanced infection control measures [11].

In the 2010 IDSA/SHEA update, there was more recognition of community‐acquired CDI and cases in long‐term care facilities. In these guidelines, a two‐step diagnostic approach was implemented by initial screening for glutamate dehydrogenase (GDH) assay, then confirming with toxin assay for GDH‐positive samples. GDH is an enzyme produced by both toxigenic and nontoxigenic *C. difficile* strains, making it a highly sensitive marker for the presence of the organism. The toxin assay for GDH‐positive samples was more specific for diagnosing active CDI. This approach was more time and cost‐efficient. Oral vancomycin became the treatment of choice for severe CDI, while metronidazole continued to be recommended for mild to moderate cases. Antibiotic stewardship and infection control measures gained recognition with emphasis on contact precautions for CDI for the first time. Fidaxomicin was approved for CDI by the FDA in 2011 [12].

Following the course of IDSA/SHEA, the 2013 ACG guidelines recommended the empirical management of CDI based on clinical suspicion, without waiting for laboratory confirmation. The guidelines recommended the nucleic acid amplification test (NAAT) as the preferred method in the diagnostic approach. Metronidazole was the first line for mild‐to‐moderate cases, and vancomycin was considered if metronidazole failed after 5–7 days. In severe cases, oral vancomycin was recommended. Fecal microbiota therapy (FMT) in these guidelines was first introduced for the third recurrence of CDI [13].

In 2017, IDSA/SHEA guidelines highlighted the growing burden of CDI, particularly in elderly and immunocompromised patients, and the emergence of hypervirulent strains like BI/NAP1/027. The guidelines recommended polymerase chain reaction (PCR) as the primary diagnostic approach. Metronidazole was now only reserved for mild cases, and fidaxomicin was introduced as an alternative treatment for moderate to severe CDI, especially for recurrent infections. The use of FMT for recurrent CDI was strongly recommended. Bezlotoxumab was first introduced as an adjunct for recurrent CDI in high‐risk patients but was not widely used or implemented [14]. The 2021 IDSA/SHEA and ACG guidelines represent the most current recommendations in the field. IDSA/SHEA now recommends oral fidaxomicin as first‐line therapy for initial and recurrent CDI, with vancomycin preserved as an acceptable alternative for both. They continue to strongly recommend FMT for recurrent diseases, as in 2017, with further emphasis on antibiotic stewardship and infection control measures [15].

Along with IDSA/SHEA, ACG again reinforced early treatment of highly suspected cases without waiting for laboratory confirmation. Vancomycin was proven more effective than metronidazole in achieving a cure, while fidaxomicin was found to be more effective than vancomycin in reducing recurrence rates. Both vancomycin and fidaxomicin were now first line for nonsevere CDI. Metronidazole use was restricted to low‐risk patients under 65 years of age in a cost‐sensitive setting [16]. FMT was now recommended from the second recurrence onward to prevent further recurrence [17]. FMT was shown to reduce the risk of CDI complications such as colectomy and sepsis when used in severe or fulminant CDI for patients who did not respond to antibiotics or were poor surgical candidates. The use of sequential FMT combined with antibiotics showed maximum efficacy in treating recurrent CDI [18].

The steady decline in CDI mortality starting around 2010, shown in our results, coincides with the updated IDSA/SHEA guidelines in 2010, ACG in 2013, and the FDA approval of new medications, as detailed above. This is likely a result of our deeper understanding of the disease’s epidemiological trends, the emphasis on initiating empiric therapy while awaiting laboratory confirmation, and the use of vancomycin, fidaxomicin, and FMT rather than metronidazole, along with more focus on antibiotic stewardship and the implementation of stricter infection control measures.

Regarding sex, there was a higher total number of deaths in females (58.65%) than males (41.35%), with an AAMR slightly higher in males throughout the study period (Figure 1 and Supporting Information 1: S1). Multiple studies have shown varying sex disparities in CDI incidence and prevalence, but the male sex has been previously linked to more severe or complicated CDI. Although the exact reasons remain unclear, this may be due to male patients having a higher likelihood of comorbid conditions that increase their risk of CDI or due to a higher use of antibiotics [19–21].

It came as no surprise that mortality rates increased with age, especially in individuals aged 65 years and older. This is consistent with previous studies that show older adults are at higher risk for severe outcomes of CDI (Figure 2). This may be due to weaker immunity, more frequent use of antibiotics, and the higher prevalence of chronic health conditions in this age group [22, 23].

Racial disparities were clear as well, showing that White individuals had the highest AAMR (6.1), followed by African Americans (5.1), American Indians/Alaska Natives (5.0), Hispanic/Latinos (3.0), and Asian/Pacific Islanders (2.3) (Figure 3 and Supporting Information 1: S2). Although the cause is not completely clear, these higher mortality rates observed in White and African American populations may be reflective of a combination of factors, including socioeconomic status, access to healthcare resources and more use of the antibiotics, and the presence of underlying health conditions [24, 25].

Geographically, the Northeast region exhibited the highest AAMR and the South region the lowest. States such as Rhode Island and Ohio had the highest mortality rates, and states like Hawaii and Louisiana had the lowest (Figures 4, 5, and 6). A possible explanation for these findings is regional differences in demographics, healthcare infrastructure, antibiotic prescribing practices, and infection control measures implemented in these regions [26]. Although not completely correlating with our results, the CDC 2020 antibiotic stewardship rates have shown significant differences in implementing these core elements among states across the United States [27].

We observed significant differences in the CDI‐related AAMR between urban and rural regions. From 1999 to 2011, trends towards higher mortality rates were noted in urban areas as compared to rural areas. Urban areas experienced an APC in the AAMR that consistently declined, while AAMR’s AAPC in rural areas steadily increased until 2015. In 2020, rural regions had a higher CDI‐related AAMR compared to urban regions. There are multiple reasons behind this trend. Firstly, rural areas are often underserved in terms of healthcare resources and infrastructure. Secondly, strict policies and precautionary measures of infection control were more rapidly implemented and enforced in urban healthcare settings, while their application in rural areas may have progressed at a slower pace [28, 29].

CDI‐related deaths occurred mostly in hospitals (78.8%), followed by nursing homes (17%) and hospice facilities (4.6%) (Figure 8). This distribution is reasonable, given that CDI is most associated with healthcare settings, particularly hospitals and long‐term care facilities [30]. The high proportion of deaths in hospitals reflects the severe and often acute nature of CDI‐related complications, such as sepsis or toxic megacolon, which frequently require hospitalization. The significant number of nursing home deaths highlights the vulnerability of residents in these facilities to healthcare‐associated infections, underscoring the need for rigorous infection control practices in long‐term care settings [31, 32].

Our study is subject to several limitations, initially directly related to its retrospective observational design, meaning the inability to establish causation with only associations reported. The nature of the CDC WONDER, which depends on death certificates, may be subject to human errors, misidentification of the cause of death, or data loss, resulting in underreporting of CDI‐related mortality. The database may lack important variables that could influence outcomes, such as socioeconomic status, healthcare access, comorbidity burden, or medical treatments used. Despite these limitations, this study sufficiently demonstrates the demographics of CDI in the United States over the past . It provides valuable insights into the effectiveness of contemporary CDI management strategies and guideline evolution.

## 5. Conclusion

Clostridioides difficile infection remains a public health concern with an ongoing burden. Our study has confirmed the decreased mortality related to the disease, which can be attributed to advances in guidelines and management protocols. We demonstrated significant differences and variations in CDI mortality with respect to sex, age, race, and geographical location. Continued efforts to improve our understanding of this disease and its trends, particularly in high‐risk populations such as the elderly and racial minorities, are essential to further reduce CDI‐related mortality in the United States.

## Author Contributions

Mohammad Kloub: literature review, manuscript writing and editing, and submission. Abdul‐rahman I Abusalim: literature review and manuscript writing. Muhammed Umer: literature review and manuscript writing. Mohamed H. Eldesouki: methods, analysis, and results. Mohamed Ahmed Ali: methods, analysis, and results. Ahmed Yasser Shaban: methods, analysis, and results. Ahmed Salem: literature review and manuscript writing. Anas Almoghrabi: literature review and manuscript writing. Jihad Slim: literature review, revision, and supervision. Yatinder Bains: literature review, revision, and supervision. Theodore Dacosta: literature review and editing.

## Funding

No funding was received for this study.

## Disclosure

The manuscript has been read and approved by all the authors.

## Consent

This study utilized publicly available, de‐identified population‐level mortality data from the CDC WONDER database. As no individual patient data were collected or accessed, institutional review board (IRB) approval and informed patient consent were not required.

## Conflicts of Interest

The authors declare no conflicts of interest.

## Supporting information


**Supporting Information** Additional supporting information can be found online in the Supporting Information section. Supporting Information. The following supplementary materials will be published alongside the article. Figure S1: Annual percentage changes in age‐adjusted mortality rates of *Clostridioides difficile* infections between 1999 and 2020 across genders. Figure S2: Annual percentage changes in age‐adjusted mortality rates of *Clostridioides difficile* infections between 1999 and 2020 across different races. Figure S3: Annual percentage changes in age‐adjusted mortality rates of Clostridioides difficile infections between 1999 and 2020 across different census regions. Figure S4: Annual percentage changes in age‐adjusted mortality rates of *Clostridioides difficile* infections between 1999 and 2020 in urban and rural areas.

## Data Availability

The datasets analyzed during the current study are publicly available for this study and can be accessed on the CDC WONDER website (https://wonder.cdc.gov)
