# Supplementary figures and images for: Trends and Disparities in Clostridioides difficile Infection Mortality in the United States From 1999 to 2020: A Nationwide Perspective
Source: Gastroenterol Res Pract. 2026 May 12;2026:9981233. doi: 10.1155/grp/9981233 (PMC13162000; doi:10.1155/grp/9981233)

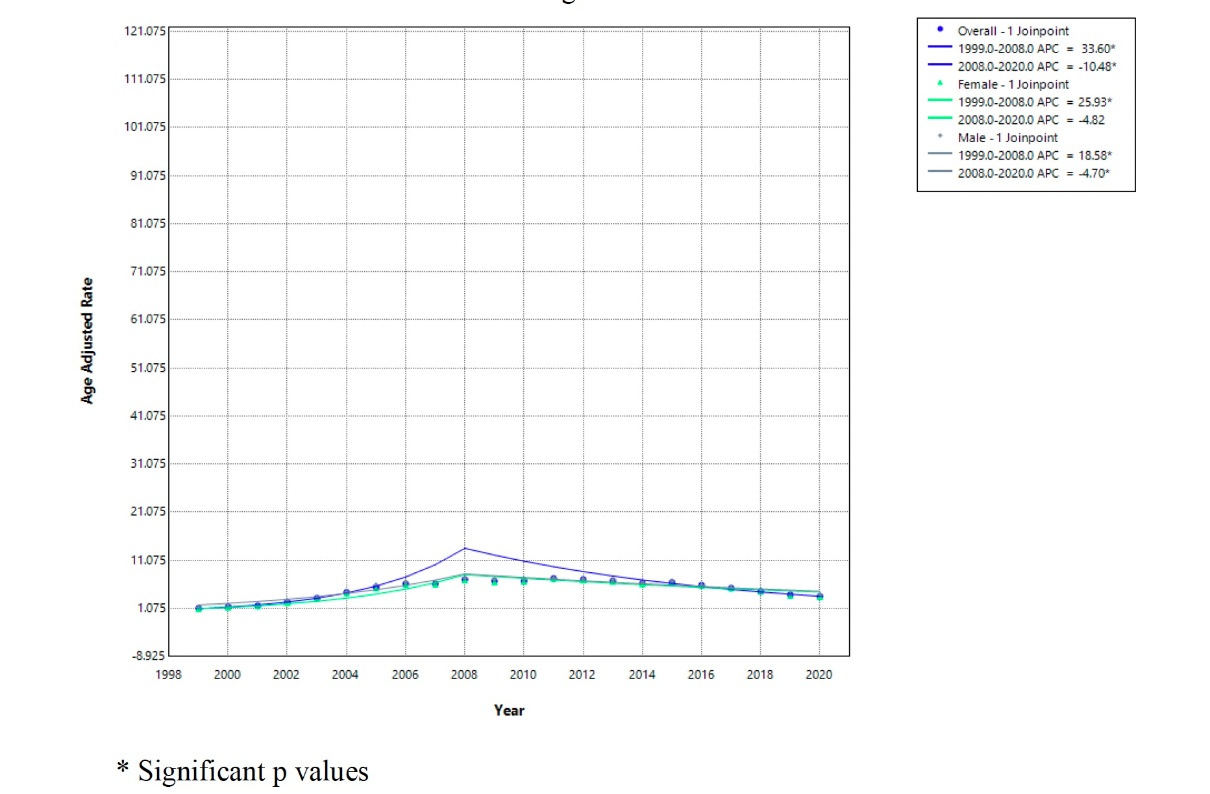


Supplementary Figure 1


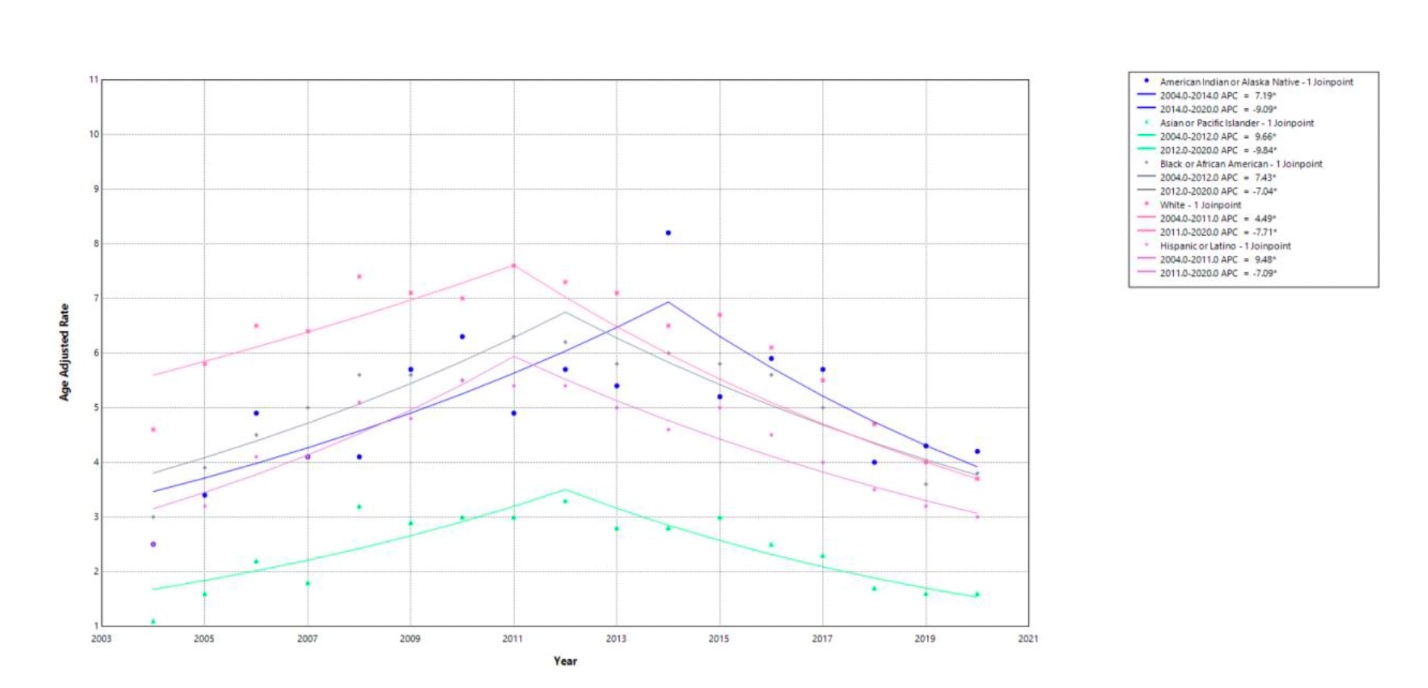


Supplementary Figure 2


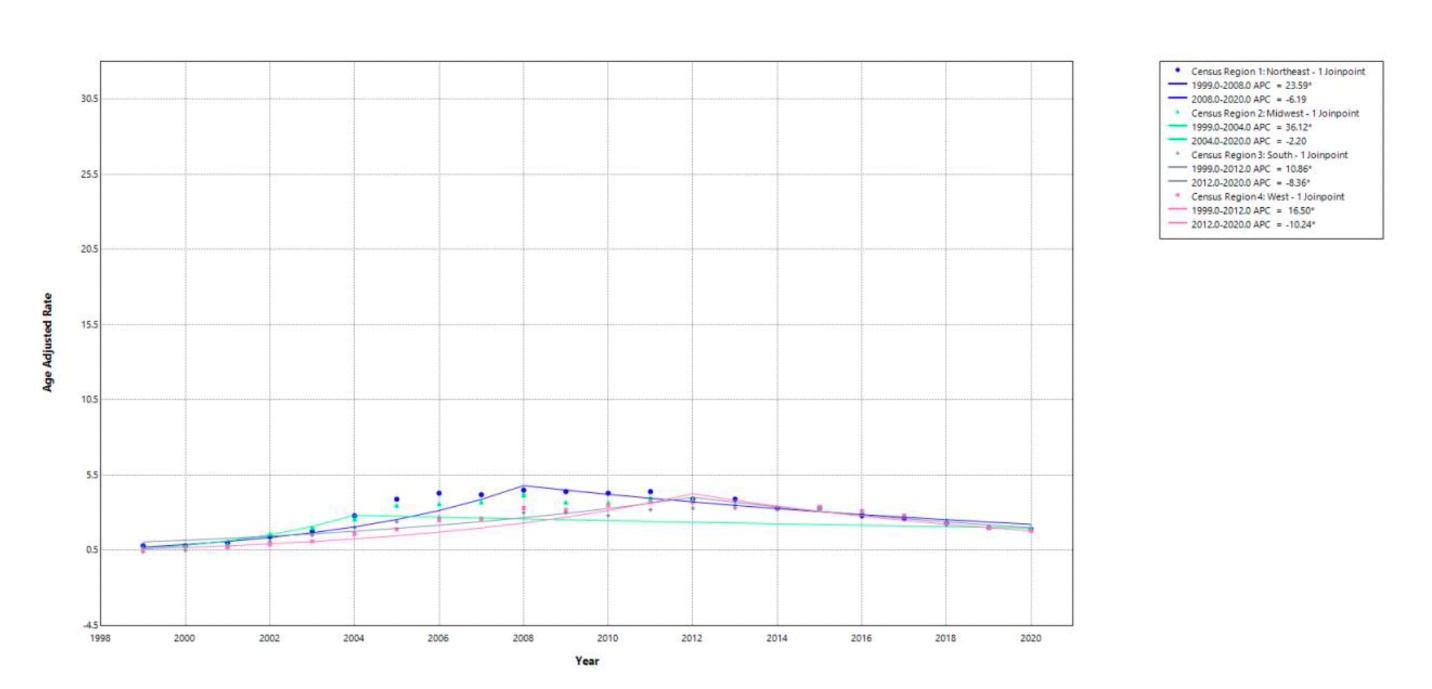


Supplementary Figure 3


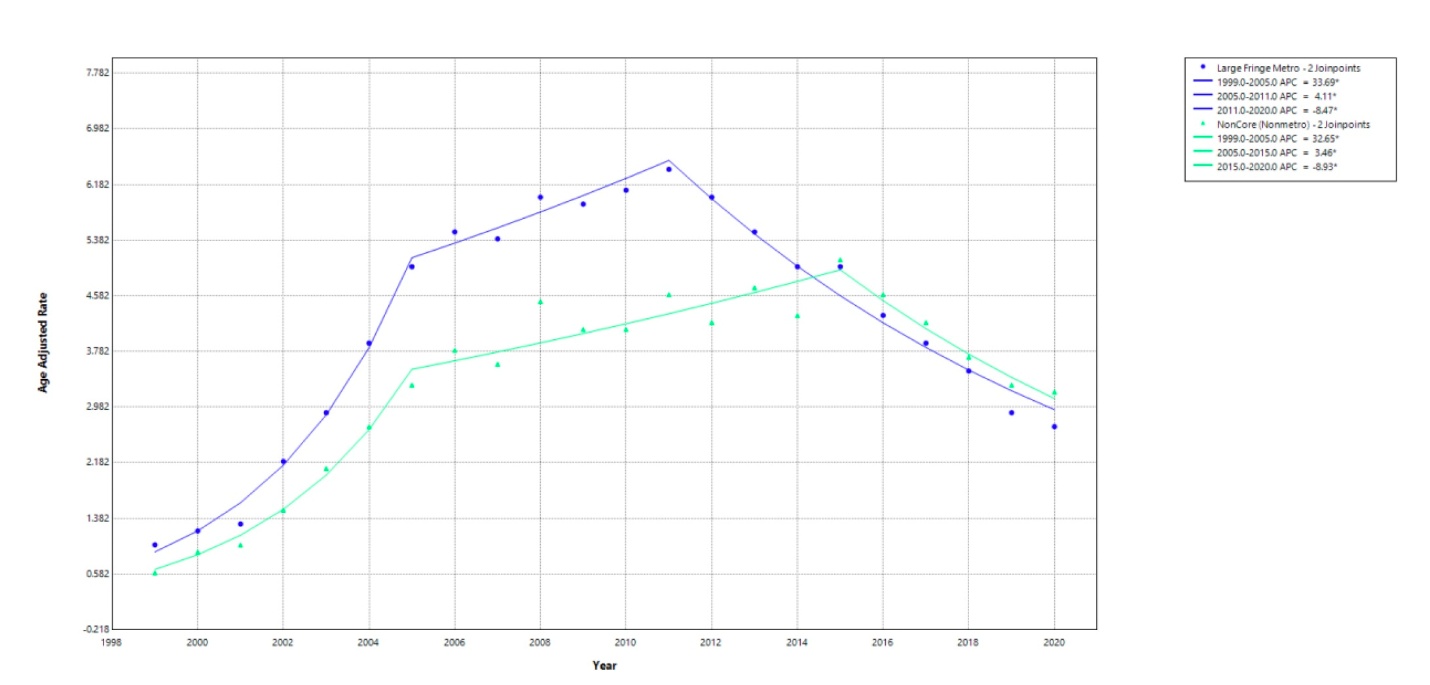


Supplementary Figure 4

Supplement: Supplementary file 1 — Supporting Information Additional supporting information can be found online in the Supporting Information section. Supporting Information. The following supplementary materials will be published alongside the article. Figure S1: Annual percentage changes in age‐adjusted mortality rates of Clostridioides difficile infections between 1999 and 2020 across genders. Figure S2: Annual percentage changes in age‐adjusted mortality rates of Clostridioides difficile infections between 1999 and 2020 across different races. Figure S3: Annual percentage changes in age‐adjusted mortality rates of Clostridioides difficile infections between 1999 and 2020 across different census regions. Figure S4: Annual percentage changes in age‐adjusted mortality rates of Clostridioides difficile infections between 1999 and 2020 in urban and rural areas. [file GRP-2026-9981233-s001.docx]
